# Multi-Focus Image Fusion: Algorithms, Evaluation, and a Library

**DOI:** 10.3390/jimaging6070060

**Published:** 2020-07-02

**Authors:** Rabia Zafar, Muhammad Shahid Farid, Muhammad Hassan Khan

**Affiliations:** Punjab University College of Information Technology, University of the Punjab, Lahore 54000, Pakistan; rabia.zafar@pucit.edu.pk (R.Z.); hassankhan@pucit.edu.pk (M.H.K.)

**Keywords:** image fusion, pixel-level image fusion, spatial domain, transform domain, quality assessment matrices

## Abstract

Image fusion is a process that integrates similar types of images collected from heterogeneous sources into one image in which the information is more definite and certain. Hence, the resultant image is anticipated as more explanatory and enlightening both for human and machine perception. Different image combination methods have been presented to consolidate significant data from a collection of images into one image. As a result of its applications and advantages in variety of fields such as remote sensing, surveillance, and medical imaging, it is significant to comprehend image fusion algorithms and have a comparative study on them. This paper presents a review of the present state-of-the-art and well-known image fusion techniques. The performance of each algorithm is assessed qualitatively and quantitatively on two benchmark multi-focus image datasets. We also produce a multi-focus image fusion dataset by collecting the widely used test images in different studies. The quantitative evaluation of fusion results is performed using a set of image fusion quality assessment metrics. The performance is also evaluated using different statistical measures. Another contribution of this paper is the proposal of a multi-focus image fusion library, to the best of our knowledge, no such library exists so far. The library provides implementation of numerous state-of-the-art image fusion algorithms and is made available publicly at project website.

## 1. Introduction

Cameras usually have limited focusing capabilities. These limitations exist due to the limited depth of field (DOF) of the optical lenses of traditional cameras. Limited DOF means that the cameras can focus a particular area and the rest of the scene remains unfocused [1,2]. Entities that are at a certain interval or in focus of the camera are captured clearly and sharply, but the objects that are in front or behind the focus of the camera lens remain blurry [3,4,5]. However, in many fields e.g., medical imaging, geographical imaging, remote sensing, and image transmission [6,7,8,9,10,11], there is a need of such images that are clear and sharp so that the interpretation and the analysis of images for different purposes can be done more efficiently and effectively. Image fusion can be used to merge multiple images captured from same or different modalities to gather additional information. For example, in medical, some tests provide information about the bony structure and some about the tissues of a certain organ, but it will be helpful if the doctor can have a single image that describes both functional and anatomical information which can be used for assessment and to plan surgical procedure [12,13]. Similarly, radiologists prefer to use integrated images for diagnostic and treatment of cancer.

In remote sensing, a remote location is analyzed and examined by satellite e.g., to estimate or detect damages in an area that is exposed to an earthquake. The equipment used does not provide convincing data. The studies have established the fact that image processing in different fields also needs images with both high spatial and spectral resolution [14,15,16]. Hence, images captured from different satellites, such as SPOT PAN, LANDSAT are fused to generate an image with high resolution [16,17,18,19]. When camera types come into consideration, each type provides us images with different information such as the image captured by infrared camera offers information that lies in the infrared spectrum and digital color camera includes information that lies in the visible spectrum. Both of these sensors complement each other’s information, e.g., in surveillance applications, a better assessment can be done with image having both type of information. Hence, merging of images can be done for effective analysis and to have the better understanding of situation [20,21,22]. The fused images not only overcome the focus constraint, they can also be easily interpreted by both human and machine.

**Figure 1:**
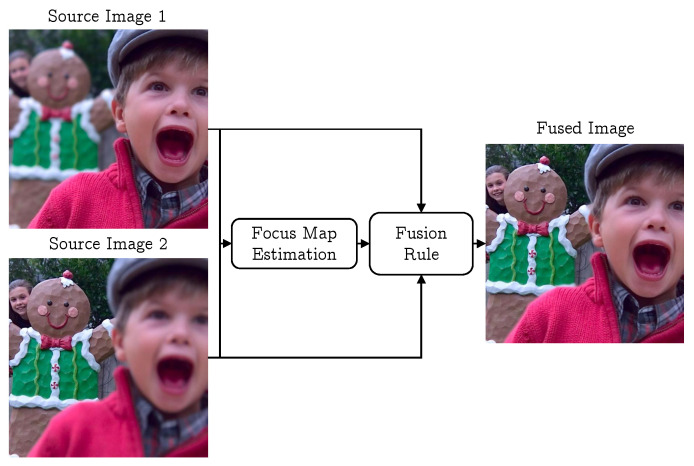
General image fusion process using two multi-focus images.

Image fusion not only makes interpretation better for the machine and human but also reduces the image transmission cost [6,23,24]. This reduction can be achieved by fusion, as after that there is no need to transmit multiple images of the same scene having the different part in focus. There will be a single image that is all-in-focus. A general multi-focus image fusion algorithm estimates a focus map for each input image. This focus map categorizes each pixel in the image as focused or defocused, discretely or continuously. The maps are used to define a fusion rule which is responsible for creating an all-in-focus image. A block diagram of such an algorithm is shown in Figure 1.

Multi-focus image fusion has received significant research efforts lately resulting in the proposal of numerous techniques e.g., [25,26,27,28,29,30,31,32]. In this paper, we review the recent literature on multi-focus image fusion and evaluate their performance visually and objectively. The performance of image fusion quality assessment metrics is also evaluated by computing different correlations. Moreover, we also present a library which offers the implementation of 24 well-known multi-focus image fusion algorithms.

The rest of the paper is organized as follows. In Section 2, we describe the criteria of effectiveness of an image fusion algorithm and group the existing techniques into different categories. Transform domain based image fusion techniques are reviewed in Section 3 and spatial domain based approaches are discussed in Section 4. The objective evaluation and the visual comparison of the results of compared methods are presented in Section 5. Section 6 introduces the proposed multi-focus image fusion library, and Section 7 draws the conclusions of this research.

## 2. Image Fusion Approaches and Criteria of Effectiveness

There are different algorithms to perform fusion, but fusion technique that needs to produce effective results should satisfy the following conditions as recommended in [24,33,34]:

- The relevant information of the source images should remain preserved.
- There should not be any inconsistencies in the fused image.
- The noise and irrelevant information should be removed or minimized as much as possible.

Numerous image fusion methods have been proposed in the recent years. Based on their representation, they are categorized into two main groups: spatial domain and transform domain [20,35,36]. The spatial domain methods involve direct execution of any calculation or procedure on the original value of the pixel. The spatial domain fusion is achieved by using localized spatial features such as pixel or regions of images [32]. The fusion procedure in spatial domain includes the combination of pixels or features that depict focused parts of the source images. Focus measures such as the energy of Laplacian or spatial frequency are used to make decisions about the focused parts of the image. We further categorized the spatial domain methods by different image representations and processing levels into three classes: pixel level fusion [28,31,36], feature level fusion [37,38], and decision level fusion [39].

**Figure 2:**
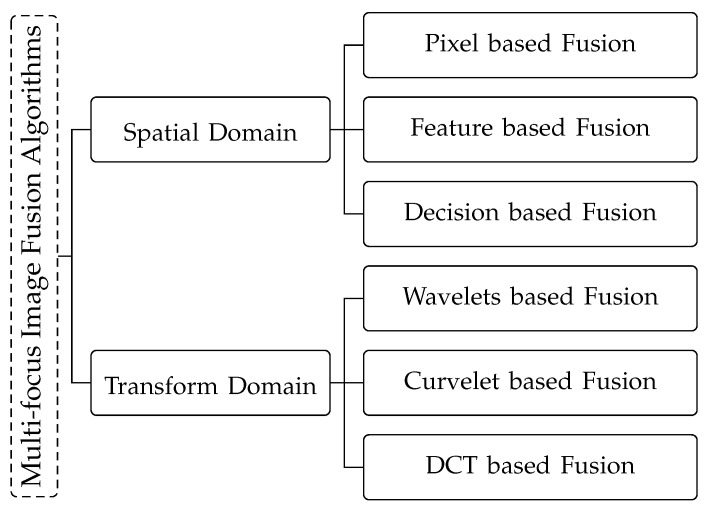
Categorization of multi-focus image fusion algorithms. The algorithms are divided into two main categories: Spatial domain based algorithms and Transform domain based algorithms, which are divided into further sub-categories. Spatial domain based algorithms: Pixel based Fusion [20,34,45,46,47,48,49,50,51,52,53,54,55,56,57,58,59,60], Feature based Fusion [21,24,33,61], Decision based Fusion [39,62,63,64]. Transform domain based algorithms: Wavelets based Fusion [24,40,44,56,65,66], Curvelet based Fusion [18,44], DCT based Fusion [22,42,67,68].

The transform domain based image fusion involves conversion of source images into transformation coefficients [18,32]. These coefficients are than fused together to get the fused image coefficients which are given to inverse transform to get resultant image. Wavelets transform [29,40,41], discrete cosine transform [42], etc. are some example of transform domain fusion. Researchers moved from spatial domain to transform domain because the latter is considered to represent the salient features of images clearly and accurately as compared to former one. However the transform domain fusion also follows the categorization as that of spatial domain fusion—pixel, feature, and decision level fusion. In the sense of its implementation, it is more significant to classify them on the basis of the transform used in fusion e.g., wavelets transform based image fusion [28,37,38,43], curvelet transform based fusion, and discrete cosine transform based image fusion [44]. Figure 2 shows our categorization of multi-focus image fusion algorithms in to different groups.

## 3. Multi-Focus Image Fusion in Transform Domain

Transform domain based multi-focus image fusion involves the conversion of source images into transformation coefficients and after applying fusion procedure the image is converted back to its own space. We broadly categorized the transform based image fusion techniques into three groups: wavelets based, curvelet based, and DCT based fusion.

### 3.1. Wavelets Based Image Fusion Techniques

The wavelet transform has been extensively explored for image fusion resulting in the proposal of a number of algorithms. The image fusion algorithm proposed in [24] converts the source images in to smaller wavelets by using filters. The images are first exposed from low-pass and high-pass filters horizontally and vertically along-with donwsampling. The fused image is generated by averaging approximation bands $I_{LL}$ of the original images and by taking the largest coefficient of each detailed sub-band. The wavelet based statistical sharpness measure (WSSM) [65] exploits marginal distribution to extract the local content information. The input images are decomposed and wavelet coefficient distribution is generated using two-component Laplacian model. Later, the approximation subband of the fused image is generated by taking the weighted average of region entropy that is calculated for each approximation coefficient using its detail subband’s coefficient. In [66], the source images are decomposed to low and high subbands. The fused sparse vector is obtained by selecting the maximum of the sparse vectors of both images. For high pass bands, the coefficient with the maximum value is selected as fused high pass band coefficient. In Non-Subsampled Contourlet Transform method [56], for each source image low pass sub band and band-pass directional sub band coefficients are generated. Then these sub bands are exposed to fusion rules.

### 3.2. Curvelet Based Image Fusion Techniques

In [18], it is demonstrated that the wavelets may not be effective when the images do not show isotropic scaling. To this end, curvelet transform based image fusion algorithm is proposed in [44]. They claim that curvelet transform is best suited for edge representation, moreover its coefficients are not affected by noise. The source images are transformed into coefficients using curvelet transform which are given as an input to the wavelet transform. The inverse curvelet transform is applied on coefficients obtained from inverse wavelet transform to get the final fused image.

### 3.3. Discrete Cosine Transform Based Image Fusion Techniques

Discrete cosine transform (DCT) [69] is a popular mean for image fusion due to its energy compactness property. In image fusion using discrete cosine transform based laplacian pyramid (DCTLP) algorithm [42], DCT is used as a reduction function to form the Laplacian pyramid. For each level, only the first half in both directions of the source image is taken as input for each next level pyramid image. The highest pyramid level is fused using the average rule i.e., the average of both band pass images is computed to obtain the next level image. The process is repeated till the last level. In [22], the discrete cosine wavelet coefficients of a particular image are calculated, then on each of the subbands DCT is applied. After computing all coefficients, the two images are fused using pixel significance details which are computed as the ration between wavelet coefficients at each level. The DCT based image fusion is efficient and time-saving but it has some limitations, such as, blocking artifacts generation and blurriness [67]. To this end, DCT with variance is implemented for fusion in [67].

## 4. Multi-Focus Image Fusion in Spatial Domain

The spatial domain based multi-focus image fusion algorithms operate directly on the image pixels to obtain all-in-focus image. We divide these approaches in to three categories: pixel-based, feature-based, and decision-based.

### 4.1. Pixel Based Multi-Focus Image Fusion Techniques

Pixel-based methods are the lowest level of fusion techniques in which the image focused data are taken from every pixel. Many multi-focus image fusion techniques suffer from different artifacts such as ringing and misregistration of boundary pixel. The dictionary based sparse representation algorithms [34,46,47,70] perform better in such cases. Sparse representation, however, has limited adeptness in detail preservation and high sensitivity to misregistration [20]. To solve these issues, convolutional sparse representation (CSR) is proposed in [20] which computes the sparse coefficients of the whole image rather than patches. It is shift invariant which helps to improve quality in misregistered regions. The guided filtering [45] is used mostly in applications when edges are needed to be preserved. In the guided filtering fusion (GFF) of images [49], the images are decomposed into the base and detail layers by using average filters. A focus map is constructed using the saliency map computed by applying Laplacian and Gaussian low pass filters on input images.

The image fusion using matting (IFM) [50] differentiates the foreground from the background using image matting and this division is used to fuse the source images. Image matting [51] measures the transparency of foreground known as alpha matte. The cross bilateral filtering (CBF) based fusion method [52] considers both geometric and gray level properties of images for integration. In [53], quad tree decomposition is used and decision is taken based on the gradient information in each patch. Self-similarity and depth information (SSDI) approach [54] identifies the similarity between the visual articles in the images which are used to obtain the fused image.

Image orientation information (OI) and pulse-coupled neural network (PCNN) is used for fusion in [55,56], respectively, by taking the orientation information of source images as a feature. In [57], gradient based focus measure is calculated for each region and a decision map is created by coping indices of regions with greater focus. In [58], fusion is done by finding two focus measures using gradient. One is used to find exact focus parts from source images whereas other one is used to find boundary pixel values. In the fusion method in [59], the decision map is computed using pixel luminance and gradient.

### 4.2. Feature-Based Multi-Focus Image Fusion

In feature based image fusion, the features such as edge details, texture, etc. are extracted from the source images and used to construct the fused image. There are numerous feature-based multi-focus image fusion algorithms e.g., DSIFT [33]. In DSIFT, local feature descriptors are extracted using the SIFT algorithm [71]. Activity level features are computed based on the local gradient and a patch is marked as focus if the average of its coefficients is greater than the corresponding patches in the other images. The focus map is then used to perform the fusion. The principal component analysis (PCA) based image fusion [24] uses co-variance and eigenvectors to get fused images. The multi-exposure fusion method proposed in [61] estimates the fused map using local contrast, exposure quality, and spatial consistency. The method in [21] uses independent component analysis to get the fused image.

### 4.3. Decision-Based Multi-Focus Image Fusion

In decision level fusion, the source images are exposed to different algorithms who act as local decision makers e.g., genetic algorithm [39]. In this method, the edges in the source images are detected and used with genetic search to find optimum weights from set of features, mean, standard deviation, and three central moments. These features are given as input to genetic search and after multiple iterations of searching it returns two optimum weights for each image. These two optimum weights are than multiplied with respective images and added to generate the fused image. The decision-based fusion algorithms are also exploited for sensor data and biometric data fusion e.g., [62,63,64].

## 5. Performance Evaluation

In this section, we evaluate the performance of 24 mulit-focus image fusion algorithms. The list of selected methods is presented in Table 1.

The evaluation is performed qualitatively and quantitatively. In particular, we used 12 objective fusion quality assessment metrics to extensively evaluate their results. We also rank the algorithms using the Borda count technique [73,74,75] to find the top performers. Moreover, an analysis of the fusion quality assessment techniques is also presented. A time complexity analysis is also carried out to measure the overall effectiveness of the algorithm.

### 5.1. Performance Evaluation Datasets

**Figure 3:**
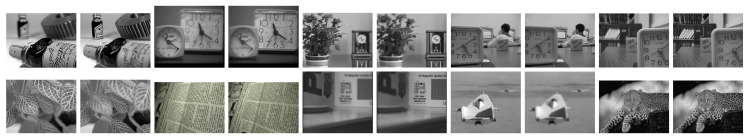
Sample multi-focus images from Grayscale dataset.

**Figure 4:**
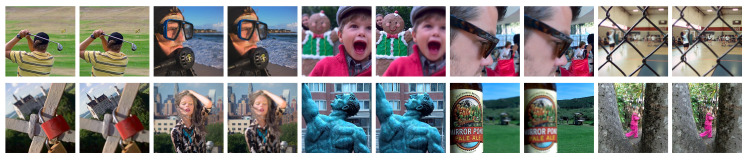
Sample images from Lytro multi-focus image fusion dataset.

The performance of MIF algorithms is evaluated on two datasets: Lytro [33] and Grayscale [60]. The former contains 20 pairs of colored multi-focus images, the dimension of each image is 520×520. Grayscale dataset consists of 11 widely used pairs of multi-focus images collected from different sources. Their dimensions vary from 160×160 to 944×736. These datasets contain indoor, outdoor, and aerial images. The image pairs are registered and each image pair has different level of details. The images in each pair are complimentary i.e., the focus region in one image is defocus in the other and vice-versa. Thumbnails of both datasets are shown in Figure 3 and Figure 4. To perform the qualitative and objective evaluations, the fused images are obtained using each algorithm on each multi-focus image set. Most of the results reported in this paper are generated from the source code provided directly by authors of the respective papers. There are only few algorithms that are coded by us or their implementations provided by third party are used. In either case, the same parameters recommended in the respective papers are used to obtain the results. Moreover, most of the algorithms do not report the results on all images or on both datasets used in our study, the results obtained on the common images are compared to verify the correctness of the implemented algorithm.

### 5.2. Qualitative Evaluation

The qualitative evaluation is performed by comparing the visual quality of the fused images. To perform this comparison, we carefully evaluated the results achieved by the compared methods on all test image pairs. However, for conciseness, we chose one representative image from both datasets and report the comparison of the results achieved by the competing algorithm. From Lytro dataset, the ‘fence’ image set is selected as the fence has cross-section areas which give us more details of edge and boundary information. From Grayscale dataset, the ‘clock’ image pair is selected which has been widely used in the image fusion literature for performance evaluation. To better express the outcomes of the visual inspection, the regions with artifacts are highlighted with red rectangles. Note that the image fusion algorithms QTD, SSDI, MSMFMg, NSCT, and OI use intensity images as input and therefore the resultant fused images are grayscale.

**Figure 5:**
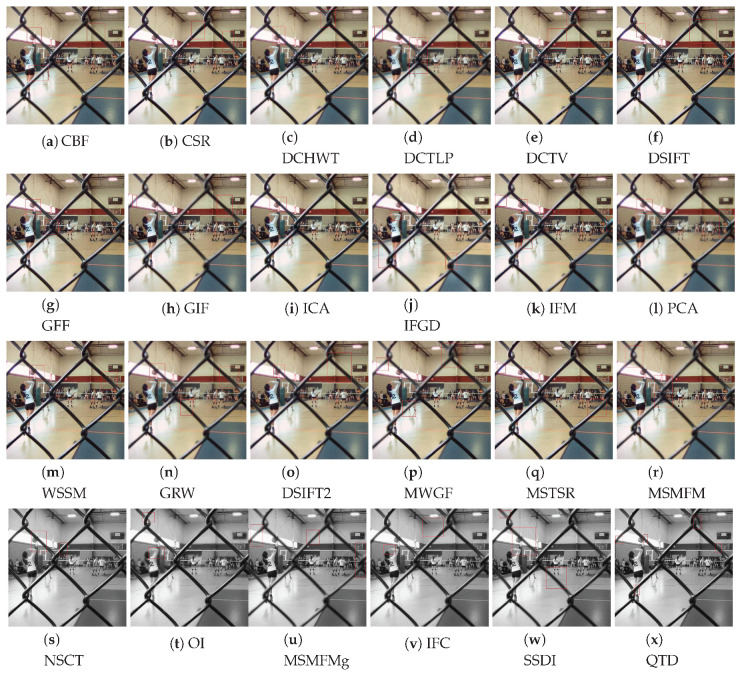
Visual comparison compared multi-focus image fusion methods on the fence image set of Lytro dataset.

Figure 5 shows the fusion results achieved by the compared methods on the fence image set. The results show that the fused images generated by MSMFM, MSMFMg, IFGD, and SSDI methods have unfocused fence regions as highlighted in Figure 5. In the results of CSR, few parts of the foreground are not sharp enough as in original images. Furthermore, the boundary of foreground and background regions are little blurred, and the color of the floor exhibits the reduction in contrast and brightness. In the DCT based approaches, DCHWT, DCTLP, and PCA, misregistration of edges is evident. The fused images look distorted, the correlation between colors also not appeared to be as agreeable as in focused part of source images. In addition to that, the ringing artifact is visible on the wall and around the boundaries of the fence.

In the fusion results of DCHWT method, the rippling artifacts appeared near the edges. Moreover, the details are not sharp, e.g., net’s pillar and people standing at the distance from the camera are indistinct-able. In case of DSIFT, in the fused image the top middle part of the fence is blurred. The foreground image is not fused well, there are solid lines, and the boundaries of focused and unfocused regions are over sharpened. The GFF method has adequate results but in this technique, fence joints are blurred and foreground image is little brighter too. However, the GFF preserves color contrast and brightness. The result of ICA exhibits blurriness near shooter’s head, near the boundaries of focused and unfocused regions and the door. The foreground is transparent near the ball. The IFM results suffer from the color distortion on the fence and the ball. Details of girl’s face are also missing. It appears that it is merged with the wall’s color and contrast.

The fused images created by the PCA algorithm are highly blurred and exhibits the ringing effect. In this example, the edges of the fence are not defined and the brightness of the image is reduced. The fusion results of WSSM method show that the edges are not fused perfectly. The fence is distorted and suffer from ghost effect at a number of regions. Moreover, the fused image has ringing effect near the wall. In the results of GIF, the fence is not clear—it is transparent and unfocused at some regions. The results of MWGF and NSCT methods still have minor unfocused regions as highlighted with red rectangles in Figure 5. The results of CBF algorithm suffer from the face over sharpening, reduction in color contrast, transparency and existence of unfocused regions at different sections. Similar artifacts can be spotted in in the fusion results achieved by the DSIFT2 and GRW methods. The results of DCTV and OI algorithms are very poor, resultant images have spherical artifacts, unfocused regions, and over sharpening of edges. The results show that the QTD algorithm performs better than other compared methods, its fusion results are free from most artifacts.

**Figure 6:**
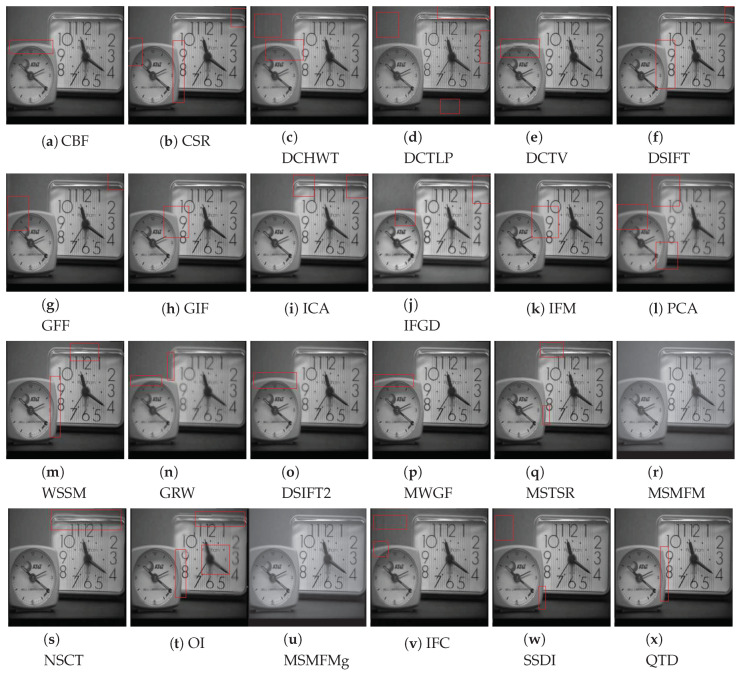
Visual comparison compared multi-focus image fusion methods on clock image set of the Grayscale dataset.

The performance of multi-focus image fusion approaches on the Grayscale dataset is discussed with the help of the clock test image pair. This image pair shows two clocks, one image shows the foreground clock in focus and in the other image the background clock is focused. The results achieved by the compared methods on this image pair are shown in Figure 6. The result shows that MSMFM and MSMFMg techniques do not exhibit any type of undecided pixel focus as well as no ghost effected area exists. Moreover, the ringing effect does not appear in the resultant images. The SSDI algorithm also shows good visual result, the only problem it faces is a small unfocused portion at the bottom left boundary of the foreground clock. A number of regions are not in focus in QTD and IFGD results. The fused image created by CSR algorithm exhibits distortion on the left boundary of the smaller timepiece. The result obtained from DCHWT approach has certain horizontal and vertical artifacts on the lower side and the upper side of the image respectively. The upper region of the clock is pixelated and similar horizontal and vertical lines are also visible.

The fused image generated by the DCTLP algorithm does not have color contrast as that exists in the original images. The DSIFT fused image exhibits good quality except few regions are grainy and has blurriness at the boundary of focused part of the foreground and the background image. The blurriness can also be spotted in the fusion results of GFF, GIF, and IFM techniques. The PCA and WSSM do not show convincing results as can be seen from Figure 6, the PCA fused image is blurry and color contrast is also not appropriate. Whereas, WSSM and MWGF exhibit structural distortion and ghost regions on the right side of the foreground clock and upper side of background clock. The fusion results achieved by CBF and MSTSR techniques suffer from color distortion and the so called grainy effect due to imperfect fusion maps. The fusion results of DCTV and OI technique are blurry and suffer from structural distortions.

From the visual comparison of the results achieved by the compared methods presented in Figure 5 and Figure 6, we conclude that considering both datasets, the visual comparisons come to the agreement that DSIFT, QTD, GFF, NSCT, and MSTSR are among the five best fusion methods for the Lytro dataset and MSMFM, MSMFMg, DSIFT, QTD, and SSDI are the best five for the Grayscale dataset.

### 5.3. Quantitative Evaluation

It is not easy to evaluate the performance of fusion algorithms. For investigation of effectiveness and evaluation of performance, two practices can be followed. First is to compare the fused image with a ground truth image (reference image), but in most practical applications, the ground truths are not available. Due to this reason the second practice came into existence, which is to assess the fused image blindly without using reference images. To validate the performance of an algorithm, along with the visual assessment the objective assessment is necessary. Therefore, numerous objective assessment models for evaluation of fusion parameters are proposed e.g., [76,77,78,79,80].

An extensive objective evaluation is performed to assess the fusion quality of the compared multi-focus image fusion algorithms. In particular, we used 12 different objective fusion quality assessment metrics in this evaluation. These metrics use various image characteristics to assess its quality and based on these characteristics they are divided in to four groups [77].

- *Information theory based metrics* measure the quality of the fused image using probability based methods i.e., mutual information, divergence, and correlation, between the fused image and the source images.
- *Feature based metrics* estimate the quality of a fused image by considering different type of features, such as, gradient, edge information, spatial frequency, etc.
- *Structural similarity based metrics* compare the structural information of the fused image and the source images to estimate the fusion quality.
- *Human perception based metrics* consider the contrast, overlapping regions, misregistration of pixel, and edge information in estimating the quality of the fused image with respect to the source images.

The metrics used in this evaluation with their respective category are listed in Table 2.

**Figure 7:**
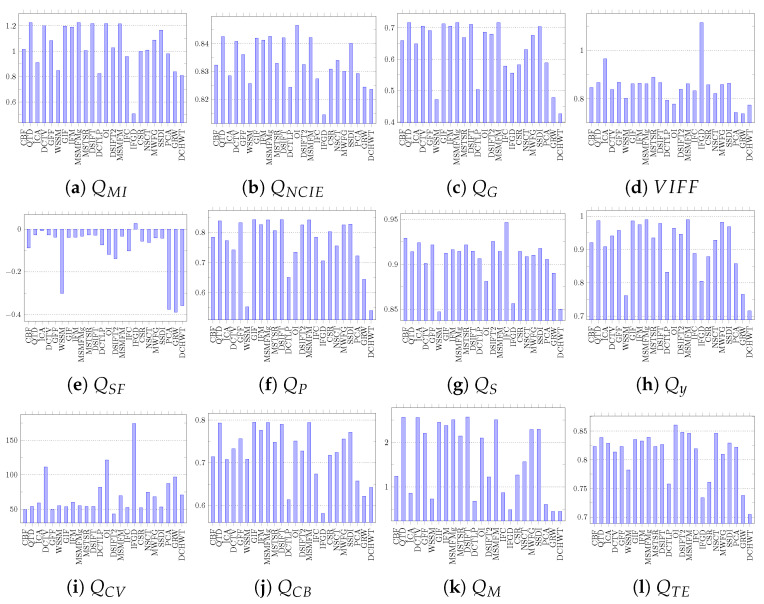
Performance of different image fusion algorithms on the Grayscale dataset using objective fusion quality assessment metrics.

**Figure 8:**
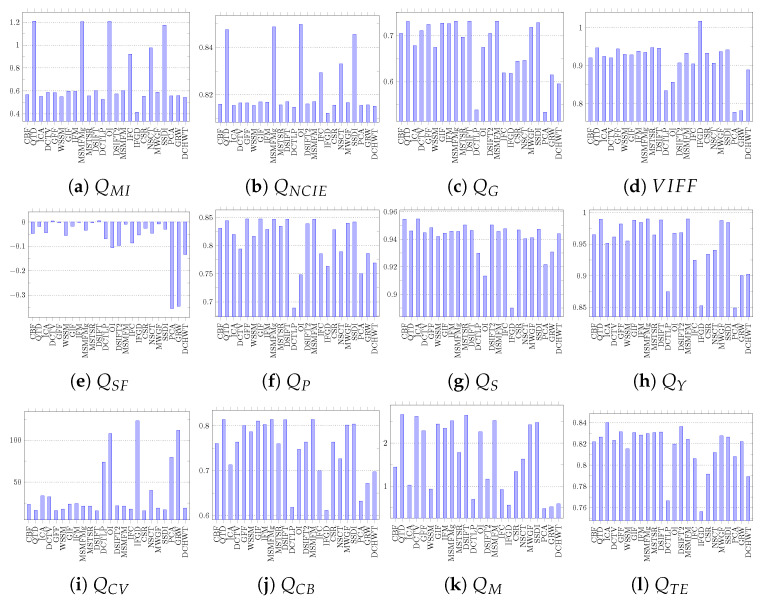
Performance of different image fusion algorithms on the Lytro dataset using objective fusion quality assessment metrics.

The fusion results obtained by each algorithm on both datasets are evaluated using all 12 objective quality assessment metrics listed in Table 2. In this evaluation, the fusion quality assessment library proposed in [77] is used. The detailed evaluation results on the Grayscale dataset are presented in Figure 7 and on the Lytro dataset are shown in Figure 8. The $Q_{MI}$ evaluates the fused images on the basis of joint and marginal probability distribution of the fused image and the source images. It ranks MSMFMg, DSIFT, and QTD as the three best algorithms for the Grayscale and QTD, MSMFM, and SSDI for Lytro. The $Q_{NCIE}$ metric takes non-linear correlation coefficient into account while evaluating the fused image. It ranks OI, MSMFM, and QTD as top algorithms for both datasets. The $Q_{TE}$ uses entropy to calculate quality of fused images and it ranks OI, DSIFT2, and NSCT on top of ranking for the Grayscale dataset. It evaluates ICA, GFF, and DSIFT2 as the top performing algorithms for the Lytro dataset. VIFF uses different models such as Gaussian scale mixture model for evaluating fused image quality. According to VIFF metric evaluation, IFGD, ICA, and MSTSR are ranked as the best performers for Grayscale whereas IFGD, MSTSR, and QTD for the Lytro dataset. The Table 3 summarizes the evaluation results based on information theory metrics and presents the best three image fusion algorithms for both datasets.

The $Q_{G}$ metric calculates edge preservation using Sobel edge detection operator as well as orientation to evaluate the fused image quality. According to this metric, MSMFMg, GIF, and QTD are amongst the best for the Grayscale dataset and MSMFM, DSIFT, and QTD for the Lytro dataset. The $Q_{SF}$ metric takes gradient information in four different directions of fused image and source images in consideration. In evaluations $Q_{SF}$ ranked IFGD, ICA, and DCTV as are the best three algorithms for the Grayscale dataset, whereas for the Lytro dataset, DSIFT, DCTV, and IFM are top ranked. The $Q_{P}$ uses the phase congruency information of the fused and source images to assess the quality. On the basis of its evaluations GIF, DSIFT, and MSMFMg are the top performing algorithms on the Grayscale dataset and GFF, GIF, and MSMFM for Lytro dataset. The $Q_{M}$ uses edge information calculated by low- and high-pass components of wavelet. The results of feature based metrics evaluation are summarized in Table 4 that shows that DSIFT, QTD, and DCTV are amongst the best algorithms for both the Grayscale and Lytro datasets. The same results are also identifiable in our visual inspection.

The structural similarity based metric $Q_{S}$ evaluates the fused image quality on the basis of variance. It ranks ICA, DSIFT2, and CBF as the best algorithms for the Grayscale dataset and ICA, CBF, and MSTSR for the Lytro dataset. The $Q_{Y}$ which takes other statistical features such as correlation, co-variance, and edge dependent information into account too while evaluating the fused image quality. It ranks GIF, MSMFMg, and QTD as the top algorithms for the Grayscale dataset and MSMFM, QTD, and DSIFT for the Lytro images. The results of structural similarity based metrics are shown in Table 5.

The summary of performance evaluation using human perception based metrics $Q_{CV}$ and $Q_{CB}$ is presented in Table 6. The $Q_{CV}$ metric considers the edge quality, similarity measurement of local regions, and global quality measurement of non-overlapping regions in evaluating the fused image quality. This metric ranks IFGD and OI as the best algorithms for both the Lytro and Grayscale datasets. The $Q_{CB}$ metric uses contrast based features to assess the fusion results. It assesses GIF, MSMFMg, and QTD as the best for the Grayscale and MSMFM and DSIFT for the Lytro dataset.

### 5.4. Borda Count Ranking of Image Fusion Algorithms

The Borda count [73,74,75] is a voting technique that ranks candidates according to voters preferences. The preferences are converted to scores and the candidate that has maximum score becomes the winner, the second highest scorer gets the second spot and so on. We use this technique here to rank the image fusion algorithms based on their ratings determined by the objective image fusion quality assessment metrics. In the present scenario, since there are 24 algorithms being evaluated, therefore an algorithm is assigned an integral value between 1 and 24 based on its performance measured by an objective quality metric. The scores are given to each algorithm in reverse proportion to their ranking. That is the best performing approach is assigned score 24 and the worst is assigned 1 score. For each fusion algorithm, these scored are accumulated to obtain an aggregated score which decides its rank.

**Figure 9:**
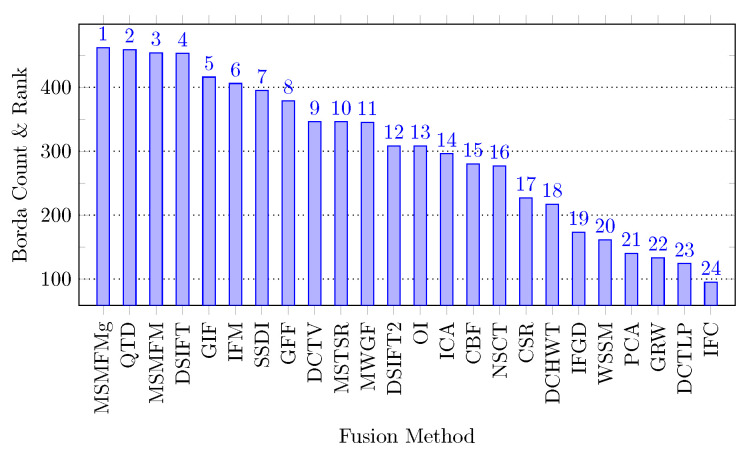
Borda count results on both dataset. The number at the column top shows its rank among the 24 compared methods.

The Borda count scores that each algorithm received on the Grayscale and Lytro datasets are presented in Table 7. The statistics reveal that for the Grayscale dataset MSMFM, MSMFMg, QTD, DSIFT, and GIF algorithms are the best five, highlighted in bold. Interestingly, these results are the same as those obtained from visual evaluation. On the Lytro dataset, the Borda count rated QTD, DSIFT, MSMFMg, MSMFM, and SSDI as the best five fusion techniques. To get an overall picture of this analysis, the scores obtained by a method on each dataset are aggregated. The algorithms are ranked based on the aggregated scores and the results are presented in Figure 9. The results show that MSMFMg is rated as the best algorithm with an aggregate score of 462, followed by QTD, MSMFM, and DSIFT with very close scores of 459, 454, and 453, respectively.

### 5.5. Summary

A summary of the findings of qualitative and quantitative evaluations is presented in Table 8. It shows that the results of both evaluations on each dataset and also an overall assessment. On the Grayscale dataset, the results of the visual and objective evaluations are the same except the visual evaluation includes SSDI where as the objective assessment brings GIF in the five best algorithms. On the Lytro dataset, the lists visually and objectively five best algorithms are the same except three differences, the qualitative list includes MSTSR, GIF, and NSCT whereas the objective list includes MSMFM, SSDI, and MSMFMg. The overall evaluation considering both datasets is the same as that of the Grayscale dataset. That is, the list of the best five algorithms contain approximately the same methods with slightly different ordering. From the statistics presented in Table 8, it can be noted that the results of the visual and the objective evaluations mostly agree, confirming the results and authenticating the effectiveness of the best rated image fusion methods.

Another interesting fact notable from Table 8 is that all the best performing methods are spatial domain based. To investigate it further, in Table 9, we list the five best performing MIF algorithms of each category with their Borda count based rank (Figure 9). The results reveal that the best eight methods among the 24 compared methods are spatial domain based algorithms. In the transform domain based methods, DCTV is the best performing and ranked at number 9 among the compared methods. Since we grouped the methods in each category into different groups, the best ranked algorithm in each group with its BC ranking is presented in Table 10. These statistics further shed light on the most suited domain/representation for efficient multi-focus image fusion. The results show that in spatial domain, the pixel based MIF algorithms are particularly performing better than other groups. Moreover, in the transform domain, the DCT based method is of particular interest. These are very interesting facts which need further investigation to discover the limitations of the frequency domain for multi-focus image fusion. We observed that the better performance of the spatial domain based methods is due to their accurate detection of focused and defocused regions which leads to crisp fusion results; such precise segmentation is not witnessed in most frequency domain methods that suffer with different artifacts e.g., ghost effect.

### 5.6. Computational Time Complexity Comparison

We also evaluate the image fusion algorithms on their computational time complexity. To this end, the execution time of each algorithm is computed on all multi-focus image pairs of the Grayscale and Lytro datasets. All algorithms were executed with the default parameters as described in the respective papers. The evaluation is performed in Matlab environment on Intel©Core™i5 processor with 4 GB RAM and 64-bit Windows 10 operating system. The execution times reported here do not include the file I/O time.

**Figure 10:**
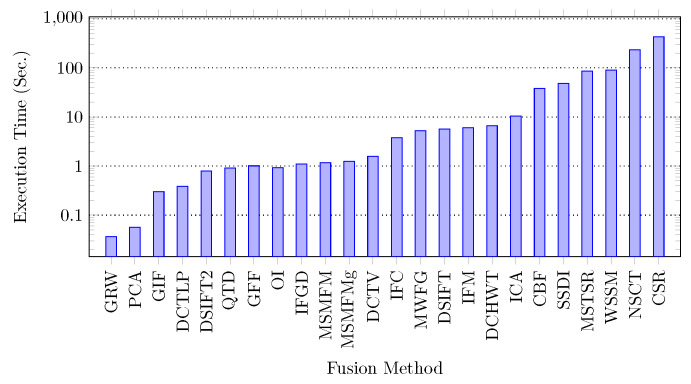
Execution time comparison of the compared methods.

For each dataset, the execution time for each image set is computed and averaged. The average execution time over both datasets is calculated for each method and the results are reported in Figure 10. To ease the analysis, the results are arranged in non-decreasing order of average time for both datasets, the bars are in blue. The results show the as many as 10 algorithms take around 1 s on average to fuse a pair of images. Seven algorithms perform fusion in average from 1 to 10 s and the other 7 methods are computationally expensive consuming 40 to 430 s per image pair. These techniques are mostly sparse representation based e.g., CSR, MSTSR, others use wavelets with pyramid e.g., WSSM which significantly increases their execution time. The method NSCT uses curvelet in combination with wavelet method and consumes more time than simple wavelet methods because of two multi-scale decomposition procedures.

## 6. Fusion Library

We also introduce a multi-focus image fusion library which provides the implementation of all the 24 multi-focus image fusion algorithms selected for evaluation in this paper, listed in Table 1. The library is implemented in MATLAB and is provided as a standalone component which contains all the dependencies, making it extremely easy to use. The current version of the proposed library supports more than 24 image fusion methods. However, this library will be kept updated by including the support for more image fusion techniques. We also encourage the multi-focus image fusion research community to provide their contributions in this field to be included in this library. The multi-focus image datasets and the library is available free at the project web-page: http://www.di.unito.it/~farid/Research/FusionLib.html.

## 7. Conclusions

The multi-focus image fusion techniques merge focused part of images of the same scene that are captured with different focus settings to obtain a single all-in-focus image. The fused image has extended depth and is effortless to be interpreted by both human and machine. The main goal of image fusion is to incorporate complementary parts of different images to get the advantageous understanding of a scenario. It increases the detail of an image and improves result’s reliability. In this study, numerous multi-focus image fusion techniques are reviewed and tested on two datasets for comparison and analysis of their performance. For evaluation of results both qualitative and quantitative approaches are considered. The second contribution of this paper is the proposal of an image fusion library. The library provides implementation of 24 image fusion methods. It is easy to use, implemented in Matlab and released free for public and peer use.

## Figures and Tables

**Table 1:** List of multi-focus image fusion (MIF) algorithms used in this study. Category represents the category of the algorithm: S for spatial and T for transform domain-based method, the the sub-category of the algorithm is provided in parenthesis.

Method	Category	Reference
CSR	S (Pixel-based)	Convolutional Sparse Representation [20]
PCA	S (Feature-based)	Principle Component Analysis [24]
DSIFT	S (Feature-based)	Dense SIFT [33]
DCTLP	T (DCT)	Discrete Cosine Transform with Laplacian Pyramid [42]
WSSM	T (DWT)	Wavelet based Statistical Sharpness Measure [65]
ICA	S (Feature-based)	Independent Component Analysis [21]
DCHWT	T (DCT)	Discrete Cosine Harmonic Wavelet Transform [22]
GFF	S (Pixel-based)	Guided filtering [49]
IFM	S (Pixel-based)	Image matting [50]
GIF	S (Pixel-based)	Guided filtering [67]
CBF	S (Pixel-based)	Image fusion based on cross bilateral filter [52]
DCTV	T (DCT)	DCT with variance [66,67]
IFGD	S (Pixel-based)	Gradient domain [59]
MSMFM	S (Pixel-based)	Multi-scale Morphological Focus Measure [57]
MSTSR	T (DWT)	Multi-scale Transform and Sparse Representation [66]
MWGF	S (Pixel-based)	Multi-scale weighted gradient-based fusion [58]
OI	S (Pixel-based)	Orientation Information and Pulse Coupled Neural Net. [55]
NSCT	T (Contourlet)	Neural Net. in Nonsubsampled Contourlet Transform [56]
QTD	S (Pixel-based)	Quadtree-based multi-focus image fusion [53]
IFC	T (DCT)	DCT Domain and Harmonic wavelet [22]
MSMFMg	S (Pixel-based)	Boundary based focus measurement [57]
SSDI	S (Pixel-based)	Fusion using self-similarity and depth information [54]
DSIFT2	S (Feature-based)	Dense SIFT for ghost-free multi-exposure fusion [61]
GRW	S (Pixel-based)	Generalized Random Walks for Fusion [72]

**Table 2:** Objective image fusion quality assessment metrics used in performance evaluation of the compared methods.

Metric Abbr.	Metric Reference
Information theory based metrics	
$Q_{M}$	Normalized mutual information [78,79]
$Q_{NCIE}$	Non-linear correlation metric [81]
$Q_{TE}$	Tselli’s entropy [82]
VIFF	Visual information fidelity metric [80]
Feature based metrics	
$Q_{G}$	Gradient based metric [76]
$Q_{SF}$	Spatial frequency based metric [83]
$Q_{M}$	Multi-scale metric [84]
$Q_{P}$	Moments based metric [85]
Structural similarity based metrics	
$Q_{S}$	Variance based metric [86]
$Q_{Y}$	Yang’s metric [87]
Human perception based metrics	
$Q_{CV}$	Chen Varshney’s metric [88]
$Q_{CB}$	Chen Blum’s metric [89]

**Table 3:** Summary of performance evaluation using information based metrics. For each metric, the numbers (1), (2), and (3) show the three best performing algorithms, respectively.

Metric	Grayscale			Lytro		
$Q_{MI}$	(1) MSMFMg	(2) DSIFT	(3) QTD	(1) QTD	(2) MSMFM	(3) SSDI
$Q_{NCIE}$	(1) OI	(2) MSMFMg	(3) QTD	(1) OI	(2) MSMFM	(3) QTD
$Q_{TE}$	(1) OI	(2) DSIFT2	(3) NSCT	(1) ICA	(2) GFF	(3) DSIFT2
VIFF	(1) IFGD	(2) ICA	(3) MSTSR	(1) IFGD	(2) MSTSR	(3) QTD

**Table 4:** Summary of performance evaluation using feature based metrics. For each metric, the numbers (1), (2), and (3) show the three best performing algorithms, respectively.

Metric	Grayscale			Lytro		
$Q_{G}$	(1) MSMFMg	(2) GIF	(3) QTD	(1) MSMFM	(2) DSIFT	(3) QTD
$Q_{M}$	(1) DSIFT	(2) QTD	(3) DCTV	(1) QTD	(2) DSIFT	(3) DCTV
$Q_{SF}$	(1) IFGD	(2) ICA	(3) DCTV	(1) DSIFT	(2) DCTV	(3) IFM
$Q_{P}$	(1) GIF	(2) DSIFT	(3) MSMFM	(1) GFF	(2) GIF	(3) MSMFM

**Table 5:** Summary of performance evaluation using structural similarity based metrics. For each metric, the numbers (1), (2), and (3) show the three best performing algorithms, respectively.

Metric	Grayscale			Lytro		
$Q_{S}$	(1) CBF	(2) DSIFT2	(3) ICA	(1) ICA	(2) CBF	(3) MSTSR
$Q_{Y}$	(1) GIF	(2) MSMFMg	(3) QTD	(1) MSMFM	(2) QTD	(3) DSIFT

**Table 6:** Summary of performance evaluation using human perception based metrics. For each metric, the numbers (1), (2), and (3) show the three best performing algorithms, respectively.

Metric	Grayscale			Lytro		
$Q_{CV}$	(1) IFGD	(2) OI	(3) DCTV	(1) IFGD	(2) OI	(3) PCA
$Q_{CB}$	(1) GIF	(2) MSMFMg	(3) QTD	(1) QTD	(2) MSMFM	(3) DSIFT

**Table 7:** Borda count results on test datasets. BC represents the cumulative Borda count score of each algorithm. The best five algorithms are marked in bold.

Metric	CBF	CSR	DCHWT	DCTLP	DCTV	**DSIFT**	GFF	**GIF**	ICA	IFGD	IFM	PCA	WSSM	GRW	DSIFT2	MWGF	MSTSR	**MSMFM**	NSCT	OI	**MSMFMg**	IFC	SSDI	**QTD**
Grayscale dataset:																								
BC	131	112	39	71	178	**227**	182	**210**	151	97	207	86	58	52	150	163	170	**237**	143	172	**238**	106	188	**232**
Rank	16	17	24	21	9	**4**	8	**5**	13	19	6	20	22	23	14	12	11	**2**	15	10	**1**	18	7	**3**
Lytro dataset:																								
BC	149	115	56	53	168	**226**	197	206	145	76	199	54	103	81	158	182	176	**217**	134	136	**224**	111	**207**	**227**
Rank	13	17	22	24	11	**2**	8	6	14	21	7	23	19	20	12	9	10	**4**	16	15	**3**	18	**5**	**1**

**Table 8:** Summary of qualitative and quantitative performance evaluation results. Only the five best performing methods are considered. In the case of quantitative evaluation, the metrics are ranked from high to low. Whereas in the case of qualitative evaluations, this ranking is not vivid—few algorithms might be indistinguishable by the visual evaluation.

Dataset	Evaluation	Methods
GS	Qualitative	MSMFM, MSMFMg, SSDI, QTD, DSIFT
	Quantitative	MSMFMg, MSMFM, QTD, DSIFT, GIF
Lytro	Qualitative	QTD, DSIFT, MSTSR, NSCT, GFF
	Quantitative	QTD, DSIFT, MSMFMg, MSMFM, SSDI
Both	Qualitative	MSMFM, DSIFT, QTD, MSMFMg, SSDI
	Quantitative	MSMFMg, QTD, MSMFM, DSIFT, GIF

**Table 9:** Five best performing MIF algorithms in each category; spatial domain and transform domain.

Category	Method	Rank
Spatial Domain	MSMFMg	1
	QTD	2
	MSMFM	3
	DSIFT	4
	GIF	5
Transform Domain	DCTV	9
	MSTSR	10
	NSCT	16
	DCHWT	18
	WSSM	20

**Table 10:** The best performing MIF algorithm in each group.

Category	Group	Method	Rank
Spatial Domain	Pixel based Fusion	MSMFMg	1
	Featurebased Fusion	DSIFT	4
Transform Domain	DCT based Fusion	DCTV	9
	Wavelets based Fusion	MSTSR	10
	Contourlet/Curvelet based Fusion	NSCT	16
